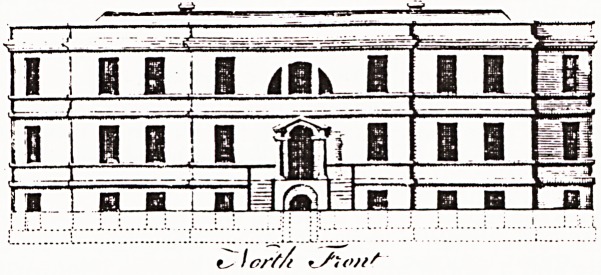# From Our Correspondents

**Published:** 1986-12

**Authors:** 


### From Our Correspondents

### The New Weston-Super-Mare General Hospital

At last a new hospital that has escaped from the 'concrete rectangular idiom' and looks as though it is meant to house humans. Consultant surgeon Tim Flew very kindly showed me around a couple of weeks after they had moved in. Built in a warm brown brick and roofed with tiles it is long and low (Figure 1), balconies and overhanging eaves suggest the Mediterranean rather than the Bristol Channel, the intimidating effect of size is reduced by a series of projecting pavilions, it is surrounded by well laid out gardens and in a few years the newly planted trees will provide greenery and shade.

The pleasant first impression is enhanced as soon as one enters the foyer beyond which is a courtyard with fountains (Figure 2) leading to the main staircase to the upper floors. Outpatients and the accident and emergency department are on the ground floor with the records department and the splendidly equipped physiotherapy and rehabilitation unit with a large gymnasium and badminton court. On the first floor are the medical wards and the pathological laboratory and on the second floor are the general surgical, orthopaedic, gynaecological, E.N.T. wards also the four operating theatres with Recovery and Intensive care wards and a Maternity Unit. There is a total of 252 beds. A long corridor with soft carpets and concealed lighting overlooks the internal courtyards and travels the length of the building with the feel of a luxury hotel rather than a hospital. Each ward has 4 six-bed units and 4 single rooms, a nursing station (Figure 3) is placed to command a view of all that goes on. A computer terminal allows information about patients to be transmited immediately from the pathological laboratory, the records department or from out patients.

Unfortunately changes of plan since the original design have already led to adaptations. After the hospital had been installed with conventionally equipped kitchens it was decided that no food should be prepared on the hospital site. Food is now prepared in the district kitchens at Brentry 30 miles away and chilled to 4 degrees and brought in bulk to Weston to be stored in refrigerators where it will keep for up to 5 days. All the new and expensive kitchen equipment has been removed and replaced by cold storage facility with microwave cookers in all the wards. Ward space originally intended for office use has been lost to 'regeneration units' for the 'cook-chill system'. When the theatres were designed the need for an endoscopy room was not envisaged and space had to be found at the expense of other use and so both the wards and the theatres are already feeling cramped and short of space. There is an Education Centre with library and a lecture room with a lounge and bar available to both doctors and the Nursing School (S.E.N.). A cluster of houses across the gardens provides a pleasant residency and there are 4 on-call bedrooms in the hospital. A cafeteria equipped with

**Figure 2**
Fountain Court and covered corridor

**Figure 3**
Nursing Station and computer terminal

**Figure 1**
The new Weston-super-Mare General Hospital microwave ovens and sophisticated refrigerated cabinets containing a variety of prepared dishes enable you to have a hot meal at any hour of the day or night. The cabinets are opened by electronic keys which have to be primed with money by being fed, along with a note into another special machine. Whether this elaborate and expensive equipment will eventually pay for itself by eliminating the wages of the domestic staff would seem doubtful and their friendly faces will surely be missed. The changeover from the General Hospital and the Royal Hospital took place with few hitches and now the settling in proces is going on.

To the casual visitor the hospital looks beautiful and equipped with the very latest technology on a lavish scale. Patients must surely be impressed. Tim Flew was pleased to have two more operating theatres (though unstaffed as yet) but sorry to have lost 11 surgical beds and have virtually nowhere to write anything on the wards, his surgical ward Sister, although she looked happy said 'Ask me again in 6 months'. I think the architect Percy Thomas and Sons of Bristol deserve our congratulations and the people of Weston-super-Mare have a lot to be pleased about. M. G. Wilson

**Plus ça Change .. The New Hospital at Taunton**

After a little matter of 40 years, Taunton's Job-like patience is being rewarded. Alongside the warren of single storey brick buildings that make up Musgrove Park Hospital (a Leggo-land vaguely reminiscent of Frenchay) has risen a magnificent new 3 storey brick building covering a couple of acres and costing £9.6 millions.

It towers over the collection of temporary buildings thrown together by the US Army Medical Corp during a few months of 1944 to take the wounded from the beaches of Normandy, and frequented (until the earth movers arrived 3 years ago) by a friendly flock of Mallard ducks who wandered in and out of the corridors.

This is Phase 1 of the Somerset Health Authority's redevelopment plan for Taunton Hospitals which will continue to put mud on our boots and dust on the bedspreads until well into the 21st century. For the first time it brings all acute medical and surgical services in Taunton onto one site. Phase 1 is particularly good news for Accident and Emergency, some of whose patients previously faced an exciting ambulance slalom across town from East Reach Hospital a mile away. A and E, Orthopaedics and Ophthalmology will share the new building with Physiotherapy, Occupational Therapy and – an innovation for West Somerset – a Hydrotherapy Unit.

Apart from a joker who slipped an eel into the hydrotherapy pool and some *opera bouffe* with the automatic bed pan washers which tend to blow when they should suck, the commissioning of the new building is going well and it should be open for businness in February 1987. The fate of the soon-to-be-abandoned East Reach Hospital has long lain in the balance. As a listed building with some striking architectural idiosyncracies it could have proved a veritable albatross to try and sell off. The Somerset Health Authority have now neatly solved that problem – as administrators do – by taking it over for their own use.

At this historic moment, Somerset greyheads who have planned and laboured for decades can afford the luxury of philosophising. A delay of decades has cost Taunton a chance to build (as provident Yeovil built) grandly and to high specifications back in the balmy '60s when land was cheap and money plentiful. More importantly perhaps, it also lost the chance to develop the country's district general hospital more strategically than on the Musgrove Park site, which is right at the Western end of the county, just as Yeovil is to the East. It has wasted hundreds of man years of frustrated efforts, grinding committee work, political lobbying, local appeals and burned candle ends. (Probably it has driven a few less than stout hearts into early retirement).

However, there are also advantages in arriving late at the party. Not for Taunton the troublesome problems of self destructing concrete which have cursed Exeter. Taunton's century-long love affair with red and orange bricks – so clearly seen in our renovated shopping centres and office blocks – continues at Musgrove Park. (Someone in the Planning Department *must* have shares.) Nor do we fool around with Babel-like tower blocks. West Somerset patients keep their feet on the ground and their heads on the second floor, and we do not propose to elevate them higher.

However, as an antidote to smugness, a physician colleague produced a magnificent copy of Toulmin's 'History of Taunton' (1791), price 3 shillings. The Tauntonians in Toulmin's day had such a high opinion of Taunton Vale that they boasted it was so fruitful 'with zun and zoil' as to need no manuring!

Recounting the assets of the 18th century county town, Toulmain describes the first General Hospital at Taunton, which was in fact the forerunner of East Reach Hospital mentioned above, and of which all but the original facade is now demolished (see figure). At that time it lay in large gardens in open countryside on the outskirts of town, a square building 90 ft long constructed around a central courtyard. All mod. cons. were present on the ground floor including a brew house, hot and cold baths, a coal pit, a 'Dead Room' and – amazingly – a laboratory. The wards and the Operation Room were on the first and second floors.

'With joy and the diffuse glow of benevolence was the foundation stone laid', recalls Toulmin, and the job was done in 1774 by no less a person than the Prime Minister Lord North. But alas, some fatal incident blasted the hopes of the afflicted. The hospital was perhaps built too large and certainly too expensively. The upkeep 'exhausted even the liberal resources and active spirit with which the undertaking commenced'. At the time of writing, 16 years later, not even one ward had been opened! Toulmin concluded sadly 'every year's neglect now tends to throw into a state of ruin an edifice which was created for the service of humanity'.

In these difficult days that tale has a chilling familiarity.

A. E. Adam.

**MRI Scanner**

*The Building Goes Up!*

The building to house the MRI Scanner at Frenchay Hospital is now in advanced stages of construction. The building work is being done by Cowlins with scaffolding by James. The whole project is being masterminded by Picker International, a branch of General Electric.

The money for the building work and funding is being raised by the Bristol MRI Scanner Fund. The machine will be the first MRI scanner in an NHS hospital in the South West.

The work is well on target for the predicted opening of scanning operations on March 1st, 1987.

The last few weeks have seen some very generous donations to the fund. The largest of these was £10,000 from the Renal Research Fund at Southmead. The consultants in the Bristol and Weston hospitals have responded generously to a letter from Dr Frank Ross with the largest contribution being £1,050 donated by Dr Russell Rees from the cardiologists research funds and from his own pocket.

A recent Fashion Show at the zoo, organised by Honey of Clifton, was a tremendous sell-out success with wonderful clothes, hats, shoes, lighting and music. The event raised around £1,500. Many thanks are due to all who took part.

The Brewer's Arms at Banwell put on a fete and had an evening of entertainment with the Doctor Jazz Quartet, raising over £700. The Anchor Inn at Bleadon put on a large auction and also entertained with Doctor Jazz. With the help of an individual donation of £500 they raised over £1,000 in one evening.

The scanner fund committee would like to thank all the people who have given up time to support the appeal. In particular they would like to thank the pubs, clubs and societies that have put on events and all the musicians who have assisted the doctors in the band.

The appeal needs £600,000 over a two year period. So far, in ten months just over £300,000 has been raised. We are therefore well on target but we are continuing in all faith that the money will be raised.

Paul Goddard

**Performance Review**

Obstetricians have long been associated with searching examinations of the causes of any maternal or neonatal child deaths occurring within their units. However culpable a doctor may have been, it is accepted that, in most cases, he has to face his colleagues and hopefully all will learn from his mistakes. Some other disciplines, too, have accepted the often painful 'death-watch' meeting where the pathologist points out the errors that physicians or surgeons have made.

Reviews of how we manage our routine consultations, admissions and operations though, are very unusual. Even the Americans find it difficult to maintain enthusiasm for their searching examinations of case reports as they seek to determine a physician's eligibility to maintain admitting rights to a hospital. In this country we do little of this. Why is it? Perhaps we are so confident in our ability to practice our particular branch of medicine that examination by our peers doesn't even enter our minds.

Perhaps it is the more likely fear of criticism or simply the fact that we are all so busy that we do not have time.

There is no doubt that examination of case reports does take an immense amount of time and effort. One other argument levelled against the proponents of review is whether it does any good; do searching reviews help change anything?

Two recent events have highlighted the issue of peer review and brought it again to the attention of the profession. The first is the 'Savage Affair' where an obstetrician was suspended from her job whilst civil lawyers examined her management of a number of deliveries, eventually deciding that she had not been guilty of professional misconduct. The second is the suggestion that general practitioners should be judged in some way to become eligible for a 'good practice allowance'. This latter suggestion was made in the government's green paper published earlier this year. The profession's response to this suggestion has not been positive, most general practitioners apparently believing that it is practically impossible to set up an efficient system that will be able to judge a general practitioner's worth.

Not all general practitioners hold these attitudes. Many nowadays enthusiastically undertake performance review. This starts within training schemes where young general practitioners bring video-recordings of their consultations for comment from their peers. They usually start apprehensively but as they realise the educational benefits that result they usually end up encouraging others to partake. Established principals, too, use video-recordings of consultations as they visit one another's practices to watch colleagues at work and make suggestions as to how improvements can be made. This is still a minority pastime but is expected to increase in frequency as the newly trained young doctors become established. Performance review by colleagues is so much more acceptable than any imposed system but, unfortunately, if the government wants to insist on its own high standards we may all have to come to terms with an imposed system. Perhaps general practice is showing the rest of the profession how best to review one another's performance and that other disciplines could well copy this approach. It is perhaps of interest that Mrs Savage has ended up based within a Department of General Practice.

M. J. Whitfield

**Care of the Elderly**

A lot of useful information is often wasted in research projects, when differing aspects of a particular problem are investigated separately by different research groups. Similar 'core' data is often collected in order to make a diagnosis or stage the severity of a disease etc. yet the criteria for so doing are not standardized. This means that very often it is impossible to draw direct comparisons between results obtained from different centres as the subject studied may vary from centre to centre. The opportunities for collaboration are also reduced for the same reasons. Although multi-centre drug trials are often regarded as unsatisfactory, especially when sponsored by a pharmaceutical company, they do at least have the merit of ensuring that there is some similarity in the subjects under investigation, although this is often far from perfect. A variation on this theme is now a step nearer within the fields of dementia research, sponsored by the Medical Research Council.

The Alzheimers Disease Research Coordinating Committee of the MRC recently established a Workshop attended by a multidisciplinary group representing most of the disciplines and centres involved in dementia research in the UK. The purpose of the Workshop was to agree the minimum information or data that should be collected on each subject in different studies into dementia funded by the Council. Without defining diagnostic criteria, the Workshop agreed on the minimum data that should be collected to allow researchers to decide whether or not a subject was likely to be demented, if so what sort of dementia they might have, and how severe the dementia was likely to be. The suggested data collection includes clinical as well as pathological studies. If the Workshop recommendations are accepted by the MRC, applicants for funding will be expected to collect this information, unless they can satisfy the independent referees and the Neurosciences Board that they should reasonably pursue a different course. It is only a beginning but it is hoped that this will make it possible for people to compare their work with the findings of colleagues elsewhere, and allow greater opportunities for collaboration. Each group will be able to interpret the data as they see fit in terms of making the diagnosis etc.

and will know that the information required for their purposes should be available in the majority of MRC funded studies.

Turning now to a different subject, the senior registrars in geriatric medicine in the South West Region have decided to get together periodically to talk about matters of mutual concern. The author was privileged to be invited to a recent meeting, when several of them presented their research projects for informal discussion. These were in varying stages of progress, ranging from those still on the drawing board to those almost ready for final presentation. It was a most enjoyable occasion, and I went away having learned a lot. They all deserve congratulations on making the time and energy available for research, in some cases on several topics, despite the majority of them being some distance away from what is normally regarded as the academic centre for the Region. It goes to show what determination and enthusiasm can achieve, as well as reflecting the high standards of geriatric departments throughout the Region.

Finally, I would like to congratulate Harbans Bhakri, currently senior registrar with Doug MacMahon and his colleagues in Truro, on being appointed to the consultant post in geriatric and general medicine at Weston-super-Mare. He will be joining Clive Bowman, and I'm sure that exciting things are going to happen over there, as they take on together the challenge of providing services for the elderly with the advent of their new hospital.

G. K. Wilcock

**The Missing Pound**

My 16 year old daughter recently returned from a sixth form course on 'management and skills training' with the following conundrum:

> Three men were staying at a small hotel, at which they were regular visitors. They spent a comfortable night and, at breakfast the next day, wished to settle their account, which was £10.00 each. They gave the waitress the money to pay their bill, which she took to the owner of the hotel.
>
> 'Ah yes,' he said, 'since they are very regular customers, I will give them a discount. Here is a £5 note for you to return to them.'
>
> On the way back to the dining-room, the waitress was puzzled as to how to return a £5 note to three equally. She looked in her pocket and found three £1 coins, so she gave the men a £1 coin each, and kept the £2 difference.
>
> So each of the men had paid £10 originally and had £1 returned, making £9 each for bed and breakfast, which is a total of £27. The waitress had kept the £2, making £29.
>
> Where is the missing £1?

Can you fault the mathematics? It was interesting to see how each member of my family dealt with the problem. The more I considered it the more I began to wonder if some of the financial arguments in the Health Service are not based on such sleight of hand. As a result of this deliberation, I wish to suggest four categories of people based on how they answer the question, 'Where is the missing pound?' They are as follows:

*Category 1.* You know *exactly* where it is and can prove it. Conclusion: You are probably a medical student (and too clever by half).

*Category 2.* You think it is wrong, haven't a clue why, so just should loudly. Conclusion: You definitely are a doctor (after all, medicine is an *art*, not a science).

*Category 3:* You wish you were the waitress. Conclusion: You are a nurse (after all, what nurse has £3 in her pocket?).

*Category 4:* You see no problem at all and are determined to proceed on that basis. Conclusion: You are a General Manager and will go far.

G. M. Stirrat

**Holidays broaden the mind and tan the body**

Travel is said to broaden the mind. I just wonder. Does travel not really widen the mind by flattening it?

Our English economic ills are too well known to recount here. In idle moments some of us think that a model of our own economic fate is clear for all to see in the lovely country of Italy. There loss of Imperial power, a sense of tradition and self-pride, combined with steady economic decline are all too clear. You only need the price of a summer holiday.

Or is that truly all? Is Europe the end of the line; what might happen if all our economies get as bad as so many gloomy prophets would have us believe?

What you obviously need now is another further-flung holiday, like say to West Africa, Abidjan or Accra have everything on offer. Holiday merchants tell no untruths. There you will find sunshine, a warm sea, palm trees, broad sandy beaches and anything else that you could dream of in a real brochure-type holiday. Body tanning is no problem.

Yet, look around. Beyond the palm trees, the beach and the bathing huts there are people who have to live all the year on the other side of the road. There, the set up is not quite so carefree. There are awful economic problems on the other side.

Economic problems? By comparison ours are paltry; non-existent, you might say. But don't get up from your swim suit, the palm trees and bathing towel, it might depress you, or merely flatten you. Just look at the social services on the other side of the road to the sand.

You can stay on the beach broadened and tanned enough not to worry.

J. D. Davies

## Figures and Tables

**Figure 1 f1:**
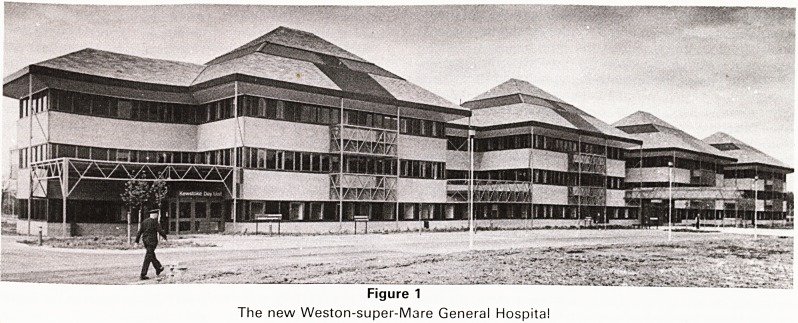


**Figure 2 f2:**
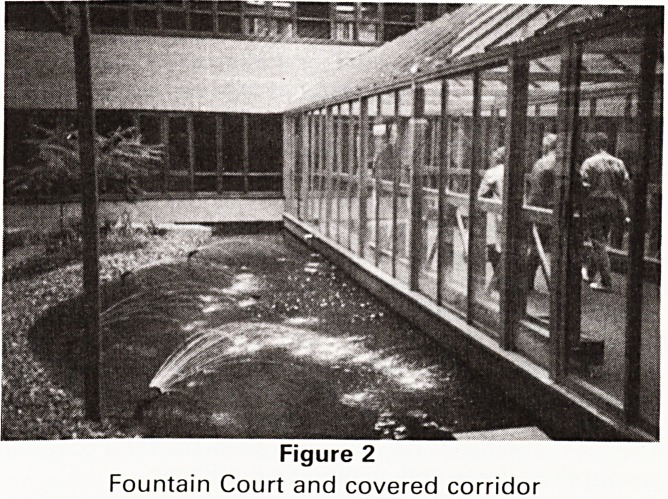


**Figure 3 f3:**
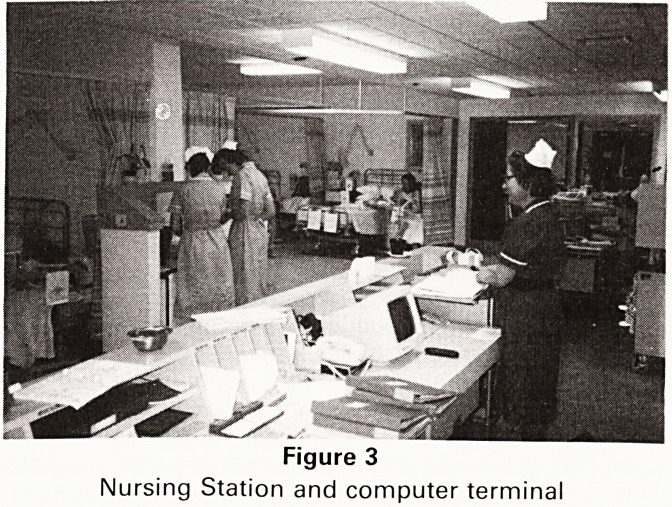


**Figure f4:**